# Clinical Outcomes in Elderly Patients with Chronic Subdural Hematoma: Validation of Irrigation Assignment Based on Hematoma Characteristics

**DOI:** 10.3390/life14040518

**Published:** 2024-04-17

**Authors:** Takuma Maeda, Yuichiro Kikkawa, Takuro Ehara, Ryosuke Tsuchiya, Shinya Tabata, Koki Onodera, Tatsuki Kimura, Yushiro Take, Kaima Suzuki, Hiroki Kurita

**Affiliations:** 1 Department of Cerebrovascular Surgery, Saitama Medical University International Medical Center, Hidaka 350-1298, Japan; 2 Department of Neurosurgery, Saitama Cardiovascular and Respiratory Center, Kumagaya 360-0197, Japan

**Keywords:** chronic subdural hematoma, elderly patients, burr hole evacuation, simple drainage, drainage with irrigation

## Abstract

The number of elderly patients with chronic subdural hematomas (CSDH) is increasing worldwide; however, there is limited data regarding the clinical outcomes in this population. Our therapeutic method using burr hole evacuation for CSDH is based on the hematoma characteristics, using simple drainage for single-layer lesions and drainage with irrigation for multiple-layer lesions. This study aimed to compare the postoperative outcomes of elderly and younger patients, identify the predictors of outcomes in elderly patients, and verify the validity of our therapeutic methods. In total, we included 214 patients who underwent burr hole evacuation between April 2018 and March 2022. Baseline characteristics, hematoma characteristics, recurrence, and clinical outcomes were compared between the elderly and younger patients. Overall, 96 elderly patients (44.9%) were included in the study, and more elderly patients underwent antithrombotic therapy than younger patients (33.3% vs. 19.5%, *p* = 0.027). Moreover, elderly patients had significantly fewer favorable outcomes than younger patients (70.8% vs. 91.5%; *p* < 0.001); however, this was not significant after adjusting for the baseline modified Rankin Scale (mRS). Similarly, elderly patients had higher recurrence rates than younger patients (10.4% vs. 2.5%; *p* = 0.021). However, the baseline mRS score was the only predictor of unfavorable outcomes. In conclusion, although the clinical outcomes of elderly patients were comparable to those of younger patients, the higher rate of preoperative antithrombotic therapy in elderly patients may result in a higher rate of recurrence requiring a long-term follow-up.

## 1. Introduction

The increasing incidence of chronic subdural hematoma (CSDH) in elderly patients is a global phenomenon driven by population aging [1]. Additionally, the increased use of antiplatelet and anticoagulant medications has significantly affected CSDH rates over the past few decades [2]. Fall injuries are considered as the most common cause of CSDH, especially in elderly patients [3]. The incidence of CSDH is currently 2–20 per 100,000 individuals, with a 3-to-1 male-to-female ratio [4]. Projections indicate that by 2030, CSDH will emerge as the predominant cranial neurosurgical condition affecting adults [2]. However, there is limited data regarding the neurosurgical outcomes in aged (≥80 years) patients with CSDH.

CSDH requires surgical treatment when a clear symptomatic or progressive increase is observed [5]. Burr hole evacuation is the most common neurosurgical procedure for patients with CSDH, regardless of age [6]. According to previous studies of patients aged ≥80 years, simple drainage during burr hole evacuation consisted of the majority of the therapeutic methods used, and irrigation was applied for all cases in only two studies [6,7,8,9,10,11,12,13]. However, no previous study has used multiple treatment methods for burr hole evacuation with or without irrigation based on hematoma characteristics. Therefore, in this study, we determined the appropriate therapeutic methods for symptomatic CSDH based on the hematoma characteristics, including simple drainage for single-layer lesions and drainage with irrigation for multiple-layer lesions. This study aimed to investigate the clinical outcomes of elderly patients with CSDH compared to those of younger patients, identify the outcome predictors, and verify the validity of our therapeutic methods.

## 2. Materials and Methods

### 2.1. Patients and Study Design

This database study was approved by the Ethics Committee of Saitama Medical University International Medical Center (approval number 17-216). We retrospectively reviewed the database of all patients (*n* = 214) with CSDH who underwent burr hole evacuation—including simple drainage and drainage with irrigation—at our institution between April 2018 and March 2022. Patients with CSDH who did not undergo surgery were excluded. The elderly group was defined as patients aged 80 years or older, and the elderly and younger groups were compared according to the following factors: age, sex, vascular risk factors (hypertension, diabetes mellitus, and hyperlipidemia), liver disease, atrial fibrillation, antithrombotic therapy including anticoagulants and antiplatelet drugs, Glasgow Coma Scale (GCS) upon admission, hematoma morphology, therapeutic modality, recurrence within 1 year (defined as a subsequent CT relapse of hematomas in the ipsilateral subdural space with any clinical presentation), and the baseline modified Rankin Scale (mRS) and discharge mRS scores.

The patients were divided into two groups based on the therapeutic modality used: simple drainage or drainage with irrigation. Patients were then divided into two further groups based on their clinical outcomes, according to the mRS score at discharge: favorable (0–3) and unfavorable (4–6).

### 2.2. Therapeutic Methods and Surgical Procedures

**Figure 1:**
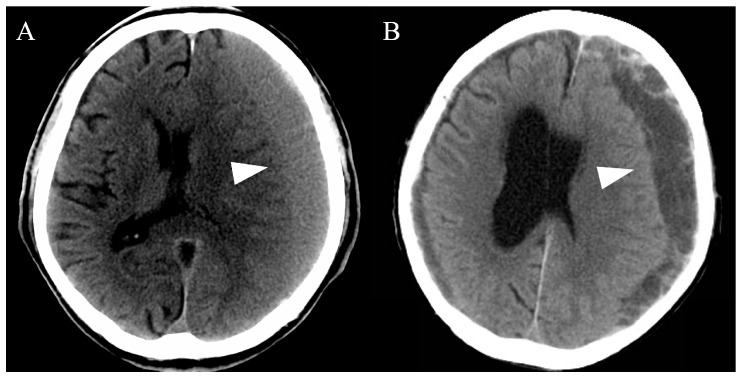
Representative computed tomography images of chronic subdural hematomas based on the hematoma morphology. All chronic subdural hematomas (arrowheads) were divided into single-layer (**A**) or multiple-layer (**B**) groups.

All cases of CSDH were detected using computed tomography (CT) and/or magnetic resonance imaging. Subsequently, we evaluated the hematoma volume and morphology, including single and multiple layers (Figure 1). Based on the CT findings, patients with a single layer underwent simple drainage, whereas patients with multiple layers underwent drainage and intraoperative irrigation with saline. After the evaluation, more than two emergency physicians and neurosurgeons discussed and determined the most appropriate therapeutic method for each patient. 

All surgical procedures were performed under local anesthesia in the operating room. Diazepam 5–10 mg and pentazocine 15 mg were injected intravenously for sedation and analgesia. After achieving a modified Richmond Agitation–Sedation Scale of ≤−3, one percent lidocaine was used for subcutaneous infiltration at the incision site. During surgery, a burr hole was placed around the center—the thickest point—of the hematoma, and after intraoperative irrigation, we placed a 5-L ventricular drainage tube anteriorly and replaced the air with room temperature saline. All patients had a subdural drain placed without being on suction or negative pressure for at least 12 h after surgery. On the first postoperative day, CT was performed, and the drainage tube was removed. If necessary, early rehabilitation was started immediately after the removal of the drainage tube.

### 2.3. Statistical Analysis

Quantitative variables are expressed as means ± standard deviations, and the x^2^ or Fisher’s exact tests were used to identify covariates that could be used as binary categorical dependent variables. For parametric data, we performed unpaired sample *t*-tests using Welch’s correction, whereas Mann–Whitney U tests were used for nonparametric data. Additionally, multivariable analysis was used to identify variables that predicted prognoses. 

All statistical analyses were performed using SPSS version 24 (IBM Corp., Armonk, NY, USA), with statistical significance set at *p* < 0.05.

## 3. Results

### 3.1. Baseline Characteristics

The baseline characteristics of the patients are summarized in Table 1. In total, 214 patients were included in this study, of which 96 (44.9%) were classified into the elderly group and 118 (55.1%) were classified into the younger group. The mean age of the patients in the elderly and younger groups was 86.4 and 70.2 years, respectively, and there were more male than female patients in both groups (64.6% vs. 72.9%, *p* = 0.051). Notably, there was a significant difference in the proportion of patients with baseline mRS 0–3 values between the elderly and younger groups (56.3% vs. 85.6%, *p* < 0.001). Furthermore, more patients had a history of hypertension (66.7% vs. 48.3%, *p* = 0.008) and atrial fibrillation (12.5% vs. 3.4%, *p* = 0.017) in the elderly group than in the younger group, and the rate of antithrombotic therapy was significantly higher in the elderly group than in the younger group (33.3% vs. 19.5%, *p* = 0.027).

The most common symptom in both groups was hemiparesis, with similar proportions between the elderly and younger groups (63.5% vs. 62.7%, *p* = 0.113). Notably, the number of patients with headaches was significantly lower in the elderly group than in the younger group (5.2% vs. 19.5%, *p* = 0.002). In contrast, the number of patients with cognitive impairment was significantly higher in the elderly group than in the younger group (32.3% vs. 11.0%, *p* < 0.001). However, other symptoms such as urinary incontinence (2.1% vs. 0%, *p* = 0.197), gait disturbance (31.3% vs. 26.3%, *p* = 0.088), and seizures (4.2% vs. 1.7%, *p* = 0.187) did not differ between the groups.

Regarding the hematoma characteristics, the mean hematoma thickness in the elderly and younger groups was 24.5 ± 5.7 (range, 7.0–38.0) mm and 23.3 ± 6.1 (range, 11.3–37.6) mm, respectively (*p* = 0.205), and bilateral CSDH was observed in 26.0% and 22.0% of the elderly and younger patients, respectively (*p* = 0.522). Notably, therapeutic modalities such as simple drainage (67.7% vs. 67.8%) and drainage with irrigation (32.3% vs. 32.2%) were similar between the groups (*p* = 0.101).

### 3.2. Clinical Outcomes

The clinical outcomes of the elderly and younger groups are shown in Table 2. Notably, significantly more patients in the younger group achieved favorable outcomes than those in the elderly group (91.5% vs. 70.8%, *p* < 0.001). However, upon subgroup analysis of the patients with a baseline mRS of 0–3, most patients in both groups achieved favorable outcomes, with no significant difference between the groups (98.1% vs. 96.0%, *p* = 0.659). Moreover, although no mortality was observed in this study, the recurrence rate was significantly higher in the elderly group than in the younger group (10.4% vs. 2.5%, *p* = 0.021). These clinical outcomes and recurrences were not affected by the therapeutic modality in each group (Table 3).

### 3.3. Predictors of Outcomes in the Elderly Patients

Table 4 presents a summary of the univariate analysis of clinical outcomes in the elderly group. We found that age, the baseline mRS score, and GCS score upon admission were significantly associated with unfavorable outcomes. Subsequently, multivariable analyses were performed to identify the predictors of unfavorable outcomes (Table 5), and logistic regression analysis showed that only the baseline mRS (odds ratio, 18.10; 95% confidence interval, 4.26–76.90) predicted unfavorable outcomes.

## 4. Discussion

Herein, we describe the characteristics of elderly patients with CSDH. We demonstrated that compared to younger patients, elderly patients (aged ≥80 years) had no significant difference in neurosurgical outcomes after adjustment for baseline mRS. According to previous literature that specifically reported on patients aged ≥80 years [6,7,8,9,10,11,12,13], mortality rates at discharge generally range from 0% to 31%, while the rates of favorable outcomes based on the mRS range from 58.8% to 100% [10,11,13]. In our study, the rates of mortality and favorable outcomes in patients aged ≥80 years were 0% and 70.8%, respectively, which is comparable to the results from previous studies.

At our institution, intraoperative irrigation with saline was performed for CSDHs with multiple layers. This differed from the therapeutic modality of previous studies on elderly patients (≥80) with CSDH, which mainly used simple drainage alone [6,7,8,9,10,11,12,13]. No previous study has provided clear irrigation criteria, and the efficacy of intraoperative irrigation remains controversial. In fact, several studies have concluded that intraoperative irrigation does not prevent recurrence [14,15], whereas others have demonstrated a preventive effect [16,17]. Additionally, some studies have reported a lower recurrence rate in patients without intraoperative irrigation [18,19]. This discrepancy may be due to the heterogeneity of the hematoma characteristics in CSDH. Intraoperative irrigation may be particularly efficacious for multiple layers of hematomas, facilitating interlayer fluid dynamics. When hematomas have multiple layers, neomembranes may resist clot evacuation through the drains because the neomembrane-forming septations prevent evacuation through the burr hole and drain, allowing the remaining clot to enlarge in the layer and eventually cause recurrence [20]. Previously, Liu et al. suggested that the treatment for CSDH should include identification and resection of abnormal membranes to avoid recurrence [21]. Craniotomy or endoscopic evacuation is generally the most appropriate therapeutic method for opening the membrane and allowing transport between multiple layers [22]; however, burr hole evacuation remains the gold standard for the first-line of treatment for symptomatic CSDH owing to its low invasiveness, operative time, and medical consumption [23], with the best scenario including breaking the membrane through a burr hole. In this study, we carefully inserted drainage tubes in several directions to achieve this goal. During this procedure, complications such as cortical bridging vein rupture, cortical damage, and hemorrhage may occur due to lack of vision during the insertion of drainage tubes or irrigation. However, in this study, we did not encounter such complications when using a 5-L ventricular drainage tube. Therefore, we believe that the selective use of irrigation is reasonable for multilayer CSDH. Currently, several randomized trials are investigating the efficacy of using drains alone or in combination with intraoperative irrigation [24,25]. These endeavors promise to elucidate the true impact of intraoperative irrigation during surgery for CSDH. 

Previous studies have reported the recurrence rate of CSDH to be approximately 10–15%, with elderly patients generally having a higher recurrence rate than younger patients [12]. Several factors induce recurrence in elderly patients, including cerebral atrophy leading to stretching of the bridging veins, increased falls, hypertension, diabetes mellitus, anticoagulant or antiplatelet use, and age-related chronic inflammation, which contributes to the development of fragile neovascular networks within the capsule [26,27]. In our study, significantly more elderly patients than younger patients received antithrombotic therapy and had a history of hypertension, which is a major risk factor for CSDH and its recurrence [26]. In fact, the risk of CSDH recurrence in patients with hypertension is approximately five times higher than that in those without hypertension [26]. Hypertension induces spontaneous intracranial bleeding, which may account for its high prevalence that increases with age in patients with CSDH. Previous studies have reported no significant association between antiplatelet therapy and CSDH recurrence when treatment was discontinued before surgery [26,28]; however, to reduce the risk of thromboembolic events, it is recommended that antithrombotic therapy is resumed as soon as possible [28]. In this study, most patients continued perioperative antithrombotic therapy to prevent thromboembolic events, which might have contributed to the higher recurrence rate in elderly patients. Therefore, further studies are warranted to investigate the appropriate management of perioperative antithrombotic therapy in CSDH. Careful and long-term follow-up may be required, especially in elderly patients with CSDH.

The present study revealed that the baseline mRS was the sole independent variable that correlated with an unfavorable outcome. In contrast, Weimer et al. [29] reported that poor neurological status at admission, age, poor premorbid functional status, a history of smoking, and perioperative fever were predictors of poor functional outcomes. Additionally, Leroy et al. [30] indicated that age, residual hematoma thickness, and a low GCS could predict clinical outcomes after surgical evacuation for CSDH, whereas sex was not associated with clinical outcomes. These disparities may be attributed to variations in the sample size, age classification, and therapeutic modalities employed. 

Although burr hole evacuation has been used in many patients, regardless of age, it is important to understand the limitations of surgical evacuation regarding the improvement in activities of daily living (ADL) in patients with CSDH. For example, in this study, 64.3% of the elderly patients with a baseline mRS of 4–5 had unfavorable outcomes after burr hole evacuation. Some deteriorations in ADL are treatable, including cognitive impairment caused by CSDH itself, also known as treatable dementia [31]. However, these symptoms are not exclusive to CSDH but are also prevalent among the geriatric population, particularly those afflicted by various medical conditions, including dementia. Determining the relative contribution of CSDH to cognitive decline is sometimes challenging; thus, careful investigations regarding the transitions of ADL and the history affecting ADL might help anticipate improvement by burr hole evacuation. Burr hole evacuation for CSDH serves not only to improve symptoms but also to save lives. Patients with CSDH are prone to falling or slipping and the bridging veins in the subdural space are stretched and thin-walled, leading to the development of an acute-on-chronic subdural hematoma [32]. In addition, CSDH usually induces excessive activation of the coagulation and fibrinolytic systems; thus, acute bleeding into the CSDH cavity may not form solid clots and easily expand [33]. Therefore, it is important to consider that acute-on-chronic subdural hematoma is not rare and is sometimes fatal.

### Limitations

This study had several limitations. First, this study operated a retrospective design in a single center, with a limited sample size. Second, there was no randomization of the therapeutic methods or absolute adaptation of a single drainage with irrigation in each case. Therefore, further studies are warranted to investigate the efficacy of both simple drainage and drainage with irrigation for the treatment of CSDH. Despite these limitations, the present study is significant because few previous reports have investigated this unique population and indicated the criteria for the irrigation of CSDH, whereas this study included a large number of elderly patients with CSDH and contributed to proposing an appropriate therapeutic method for CSDH in this aging society.

## 5. Conclusions

In this study, we found that the clinical outcomes in elderly patients with CSDH were comparable to those in younger patients after adjusting for baseline mRS when therapeutic methods based on hematoma characteristics were used. Moreover, the baseline mRS score was the only predictor of unfavorable outcomes. A higher rate of preoperative antithrombotic therapy may result in a higher rate of recurrence in elderly patients requiring long-term follow-up. It is important to understand the limitations of ADL improvement following burr hole evacuation in elderly patients with poor ADL.

## Figures and Tables

**Table 1:** Baseline characteristics of the patients.

	Aged ≥80 Years	Aged <80 Years	*p*-Value
Number of patients	96 (44.9)	118 (55.1)	
Mean age ± SD (range), years	86.4 ± 4.8 (80–98)	70.2 ± 8.7 (31–79)	
Sex (male)	62 (64.6)	86 (72.9)	0.051
Baseline mRS 0–3	54 (56.3)	101 (85.6)	<0.001
Patient history			
Hypertension	64 (66.7)	57 (48.3)	0.008
Diabetes mellitus	18 (18.8)	21 (17.8)	0.139
Hyperlipidemia	12 (12.5)	13 (11.0)	0.159
Liver disease	2 (2.1)	3 (2.6)	1.000
Atrial fibrillation	12 (12.5)	4 (3.4)	0.017
Antithrombotic therapy	32 (33.3)	23 (19.5)	0.027
GCS upon admission *			
3–8	2/85 (2.4)	3/111 (2.7)	1.000
9–12	9/85 (10.6)	3/111 (2.7)	0.033
13–15	74/85 (87.0)	105/111 (94.6)	0.089
Symptom			
Headache	5 (5.2)	23 (19.5)	0.002
Urinary incontinence	2 (2.1)	0 (0)	0.197
Hemiparesis	61 (63.5)	74 (62.7)	0.113
Gait disturbance	30 (31.3)	31 (26.3)	0.088
Cognitive impairment	31 (32.3)	13 (11.0)	<0.001
Seizure	4 (4.2)	2 (1.7)	0.187
Hematoma			
Mean maximum thickness ± SD (range), mm	24.5 ± 5.7 (7.0–38.0)	23.3 ± 6.1 (11.3–37.6)	0.205
Mean midline shift ± SD (range), mm	6.4 ± 4.9 (0–19.3)	6.6 ± 5.3 (0–19.0)	0.789
Bilateral CSDH	25 (26.0)	26 (22.0)	0.522
Therapeutic modality			0.101
Simple drainage	65 (67.7)	80 (67.8)	
Drainage with irrigation	31 (32.3)	38 (32.2)	

Values are expressed as *n* (%), except where indicated otherwise; * Sample sizes differ due to missing values; Abbreviations: GCS, Glasgow Coma Scale; CSDH, chronic subdural hematoma; mRS, modified Rankin Scale; SD, standard deviation.

**Table 2:** Clinical outcomes of the elderly and younger groups.

	Aged ≥80 Years	Aged <80 Years	*p*-Value
Favorable outcome	68 (70.8)	108 (91.5)	<0.001
Baseline mRS 0–3	53 (98.1)	97 (96.0)	0.659
Baseline mRS 4–5	15 (35.7)	11 (64.7)	0.050
Mortality	0 (0)	0 (0)	-
Recurrence within 1 year	10 (10.4)	3 (2.5)	0.021
Median time to recurrence (IQR), days	28 (6–140)	33 (18–44)	1.000

Values are expressed as *n* (%), except where indicated otherwise; Abbreviations: mRS, modified Rankin Scale.

**Table 3:** Clinical outcomes of the elderly and younger groups by therapeutic methods.

	Aged ≥80 Years			Aged <80 Years		
	Simple	w/Irrigation	*p*-Value	Simple	w/Irrigation	*p*-Value
Favorable outcome	48 (73.8)	20 (64.5)	0.350	76 (95.0)	32 (84.2)	0.074
Baseline mRS 0–3	37 (97.4)	16 (100)	1.000	67 (98.5)	30 (90.9)	0.101
Baseline mRS 4–5	11 (40.7)	4 (26.7)	0.506	9 (75.0)	2 (40.0)	0.280
Recurrence within 1 year	5 (7.7)	5 (16.1)	0.284	3 (3.8)	0 (0)	0.550

**Table 4:** Univariate analysis for favorable and unfavorable outcomes in elderly patients.

	Favorable	Unfavorable	*p*-Value
Number of patients	68 (70.8)	28 (29.2)	
Mean age ± SD (range), years	85.3 ± 4.6 (80–98)	87.9 ± 5.2 (80–98)	0.015
Sex (male)	46 (67.6)	16 (57.1)	0.355
Baseline mRS 0–3	53 (77.9)	1 (3.6)	<0.001
Patient history			
Hypertension	47 (69.1)	17 (60.7)	0.479
Diabetes mellitus	14 (20.6)	4 (14.3)	0.574
Hyperlipidemia	11 (16.2)	1 (3.6)	0.171
Liver disease	1 (3.6)	1 (1.5)	0.510
Atrial fibrillation	9 (13.2)	3 (10.7)	1.000
Antithrombotic therapy	24 (35.3)	8 (28.6)	0.636
GCS upon admission *			
3–8	0/64 (0)	2/21 (9.5)	0.063
9–12	3/64 (4.7)	6/21 (28.6)	0.008
13–15	61/64 (95.3)	13/21 (61.9)	<0.001
Symptom			
Headache	4 (5.9)	1 (3.6)	1.000
Urinary incontinence	2 (2.9)	0 (0)	1.000
Hemiparesis	39 (57.4)	22 (78.6)	0.063
Gait disturbance	27 (39.7)	3 (10.7)	0.007
Cognitive impairment	24 (35.3)	7 (25.0)	0.472
Seizure	3 (4.4)	1 (3.6)	1.000
Hematoma			
Mean maximum thickness ± SD (range), mm	24.4 ± 5.7 (11.0–38.0)	25.3 ± 5.7 (13.0–35.0)	0.488
Mean midline shift ± SD (range), mm	6.2 ± 5.5 (0–17.6)	6.9 ± 5.5 (0–19.0)	0.566
Bilateral CSDH	21 (30.9)	4 (14.3)	0.126
Therapeutic modality			0.350
Simple drainage	48 (70.6)	17 (60.7)	
Drainage with irrigation	20 (29.4)	11 (39.3)	
Recurrence within 1 year	6 (8.8)	4 (14.3)	0.471

Values are expressed as *n* (%), except where indicated otherwise; * Sample sizes differ due to missing values; Abbreviations: GCS, Glasgow Coma Scale; CSDH, chronic subdural hematoma; mRS, modified Rankin Scale; SD, standard deviation.

**Table 5:** Multivariable analysis for unfavorable outcomes in elderly patients.

	OR (95% CI)	*p*-Value
Age, years	1.07 (0.95–1.20)	0.255
Baseline mRS	18.10 (4.26–76.90)	<0.001

The area under the curve of the model is 0.913. Abbreviations: CI, confidence interval; mRS, modified Rankin Scale; OR, odds ratio.

## Data Availability

The data presented in this study are available on request from the corresponding author.
